# Machine-Learning Algorithm-Based Prediction of Diagnostic Gene Biomarkers Related to Immune Infiltration in Patients With Chronic Obstructive Pulmonary Disease

**DOI:** 10.3389/fimmu.2022.740513

**Published:** 2022-03-08

**Authors:** Yuepeng Zhang, Rongyao Xia, Meiyu Lv, Zhiheng Li, Lingling Jin, Xueda Chen, Yaqian Han, Chunpeng Shi, Yanan Jiang, Shoude Jin

**Affiliations:** ^1^Department of Respiratory Medicine, The Fourth Hospital of Harbin Medical University, Harbin, China; ^2^Department of Respiratory Medicine, The Second Hospital of Harbin Medical University, Harbin, China; ^3^Department of Medical Oncology, The Fourth Hospital of Harbin Medical University, Harbin, China; ^4^School of Instrumentation Science and Engineering, Harbin Institute of Technology, Harbin, China; ^5^Department of Pharmacology, State-Province Key Laboratories of Biomedicine- Pharmaceutics of China, Key Laboratory of Cardiovascular Research, Ministry of Education, College of Pharmacy, Harbin Medical University, Harbin, China; ^6^Translational Medicine Research and Cooperation Center of Northern China, Heilongjiang Academy of Medical Sciences, Harbin, China

**Keywords:** COPD, LASSO, SVM-RFE, STAU1, SLC27A3, immune infiltration

## Abstract

**Objective:**

This study aims to identify clinically relevant diagnostic biomarkers in chronic obstructive pulmonary disease (COPD) while exploring how immune cell infiltration contributes towards COPD pathogenesis.

**Methods:**

The GEO database provided two human COPD gene expression datasets (GSE38974 and GSE76925; n=134) along with the relevant controls (n=49) for differentially expressed gene (DEG) analyses. Candidate biomarkers were identified using the support vector machine recursive feature elimination (SVM-RFE) analysis and the LASSO regression model. The discriminatory ability was determined using the area under the receiver operating characteristic curve (AUC) values. These candidate biomarkers were characterized in the GSE106986 dataset (14 COPD patients and 5 controls) in terms of their respective diagnostic values and expression levels. The CIBERSORT program was used to estimate patterns of tissue infiltration of 22 types of immune cells. Furthermore, the *in vivo* and *in vitro* model of COPD was established using cigarette smoke extract (CSE) to validated the bioinformatics results.

**Results:**

80 genes were identified *via* DEG analysis that were primarily involved in cellular amino acid and metabolic processes, regulation of telomerase activity and phagocytosis, antigen processing and MHC class I-mediated peptide antigen presentation, and other biological processes. LASSO and SVM-RFE were used to further characterize the candidate diagnostic markers for COPD, SLC27A3, and STAU1. SLC27A3 and STAU1 were found to be diagnostic markers of COPD in the metadata cohort (AUC=0.734, AUC=0.745). Their relevance in COPD were validated in the GSE106986 dataset (AUC=0.900 AUC=0.971). Subsequent analysis of immune cell infiltration discovered an association between SLC27A3 and STAU1 with resting NK cells, plasma cells, eosinophils, activated mast cells, memory B cells, CD8+, CD4+, and helper follicular T-cells. The expressions of SLC27A3 and STAU1 were upregulated in COPD models both *in vivo* and *in vitro*. Immune infiltration activation was observed in COPD models, accompanied by the enhanced expression of SLC27A3 and STAU1. Whereas, the knockdown of SLC27A3 or STAU1 attenuated the effect of CSE on BEAS-2B cells.

**Conclusion:**

STUA1 and SLC27A3 are valuable diagnostic biomarkers of COPD. COPD pathogenesis is heavily influenced by patterns of immune cell infiltration. This study provides a molecular biology insight into COPD occurrence and in exploring new therapeutic means useful in COPD.

## Introduction

Chronic obstructive pulmonary disease (COPD) is an extremely common debilitating ailment of the modern world and represents a slowly progressive destructive pulmonary disease that involves chronic inflammation (1). COPD is currently ranked as the fourth cause of death in the world and is common in both developed and developing countries (2, 3). Early COPD diagnosis and initiation of treatment are clinically important in improving prognosis and overall survival of COPD patients. COPD is definitively diagnosed with pulmonary function testing, which offers the advantage of being a simple and easily implemented measurement that accurately gauges the severity of COPD. A wide array of imaging modalities such as chest x-rays, magnetic resonance imaging (MRI), and computed tomography (CT) have further improved COPD diagnosis (4). However, due to sensitivity and specificity limitations, current diagnostic mechanisms are not well suited in detecting early COPD. COPD develops on well-established disease drivers such as genetic factors, a history of smoking, infection, and inflammation, along with an imbalance in protease and antiprotease expression (5). COPD is indeed a complex and multifactorial entity that arises due to an amalgamation of environmental and genetic influences (6).

In recent years, the application of microarray technology and integrated bioinformatics analyses have been used to identify novel diagnostic and prognostic genes (7–11). In the realm of COPD, for example, the ROBO2 and SLIT2 genes were founded to be downregulated in COPD and inversely correlated with COPD disease stage (12, 13). FHL1 expression was associated with cigarette smoke-induced COPD (14). Additionally, increasingly evidences highlight the importance of immune cell infiltration in generation progression of various diseases (7, 15–17). However, little is known about immune cell infiltration in COPD.

This investigation involves a metadata cohort comprising of 2 COPD microarray datasets extracted from the GEO database. Differentially expressed gene (DEG) analysis was then performed between healthy samples and in COPD samples. Potentially diagnostic COPD biomarkers were discerned using machine-learning algorithms which were then validated in a separate cohort, with a logistic regression method utilizing in constructing a diagnostic prediction model. Immune cell gene expression was analyzed using CIBERSORT which successfully identified and quantified various immune cells that infiltrated into the lung parenchyma in COPD and control samples. The relationship between immune cell infiltration and identified COPD biomarkers was also characterized.

## Materials and Methods

### Data Collection and Download

COPD gene expression data collected from the GEO database (https://www.ncbi.nlm.nih.gov/geo/). The GSE38974, GSE76925, and GSE106986 datasets were downloaded. The GSE106986 dataset (14 COPD samples and 5 control samples) was derived from GPL13497 platform of the Agilent-026652 Whole Human Genome Microarray 4x44K v2. The GSE76925 dataset (111 COPD samples and 40 control samples) was derived from the GPL10558 platform of Illumina HumanHT-12 V4.0 expression beadchip. GSE38974 dataset (23 COPD samples and 9 control samples) was derived from the GPL4133 platform of the Agilent-014850 Whole Human Genome Microarray 4x44K G4112F. All samples from the 3 different databases were obtained from human lung tissue.

### Data Preprocessing and Differentially Expressed Genes (DEGs) Screening

Each dataset was background corrected and normalized using the “limma” package and converted to gene symbols referencing the probe annotation files of the probe names in each dataset. A metadata cohort comprising of merged GSE38974 and GSE76925 cohorts was created for further analysis. Batch variability between platforms was eliminated using the combat function of the “SVA” package (18). The GSE106986 dataset was used as the validation cohort. Similarly, 134 COPD and 49 normal samples were analyzed for DEGs using the R package “limma” with DEGs selected based on thresholds of *P* < 0.05 and |*log2 FC*| > 1.

### Functional Enrichment Analysis

The Metascape (http://metascape.org) database allowed for further understanding of the DEG biological significance using Gene Ontology enrichment analysis. The Molecular Signatures Database (MSigDB) Hallmark Gene Sets and Kyoto Encyclopedia of Genes and Genomes (KEGG) Pathway were used for pathway enrichment analysis. Meanwhile, the most significant terms which arose in statistical analysis were selected for visualization.

### Screening of Candidate Diagnostic Biomarkers

Two machine-learning algorithms, the support vector machine recursive feature elimination (SVM-RFE) and least absolute shrinkage and selection operator (LASSO) were used in this study to screen for significant prognostic variables. SVM-RFE represents a widely used supervised machine-learning protocol for classification and regression that is applied using the “e1071” package. The SVM-RFE algorithm was used to identify genes with higher discriminative power (19). LASSO was performed using the “glmnet” package in R and represents a regression analysis algorithm that applies regularization for variable selection. Using LASSO, we were able to identify genes significantly associated with COPD and normal samples. We applied two algorithms in the metadata cohort and utilized the GSE106986 dataset to analyze overlapping genes from the two algorithms to further validate the expression levels of candidate diagnostic biomarkers.

### Evaluation of Immune Cell Infiltration

CIBERSORT is a deconvolution algorithm that quantifies immune cell infiltration (22 various cell types) in COPD gene expression profiles (20). The “corrplot” R package was used to carry out visualization and correlation analysis of 22 types of infiltrating immune cells. Differences in infiltrating immune cells between COPD and healthy samples were visualized using boxplots drawn with the “ggplot2” package in R.

### Correlation Analysis Between Diagnostic Biomarkers and Infiltrating Immune Cells

Pearson correlation analysis allowed for in-depth scrutiny of relationships between diagnostic biological markers and infiltrating immune cells. “ggplot2” of the R package was used to visualize the results of the analysis.

### The Establishment of COPD Model

Six-week-old C57BL/6 mice was used to establish the COPD model. The details procedure was as described by He et al. (21). Cigarette smoke extract (CSE) was prepared by dissolving the smoke of non-filter Furong cigarette in to PBS (21). The histological changes and immune infiltration of lung tissues were then detected (21, 22). Bronchoalveolar lavage fluid (BALF) was collected. Lung tissue was cut into small pieces and digested into single cell suspension by Collagenase A (4 mg/ml in RPMI1640) for further detection.

### Histological Staining

After treatment, lung tissues were collected. Paraffin embeded lung tissues were cut into slices for histological staining. HE staining was performed according to the manufacturer’s instructions (Solarbio). The mean alveolar septal thickness (MAST), mean linear intercept (MLI), and destructive index (DI) were calculated. Immunohistochemistry staining was conducted using primary antibodies for SLC27A3 (Proteintech), STAU1 (Affinity), CD57 (Affinity), and CD8a (Affinity). Immunofluorescence staining was performed using primary antibodies for CD19 (Affinity) and CD27 (Santa), and secondary antibodies including Cy3-labeled goat anti-rabbit IgG (Invitrogen) and FITC-labeled goat anti-mouse IgG (Abcam).

### Cell Transfection and CCK-8 Assay

BEAS-2B cells were cultured in DMEM cultural medium (Servivebio) with 10% fetal bovine serum (EVERY GREEN) in an incubator (37°C and 5% CO_2_). COPD cell model was induced by 5% CSE for 24 h (23). Small interfering RNAs targeting SLC27A3 (si-SLS27A3) and STAU1 (si-STAU1), as well was negative control RNA (si-NC) were transfected into BEAS-2B cells by Lipo 3000 (Invitrogen), respectively. CCK-8 assay kit (Wanleibio) was used to detect viability of BEAS-2B cells according to the manufacturer’s instructions. The optical density was measured by a microplate reader (BIOTEK).

### Western Blot Assay

Total protein was extracted from lung tissues and BEAS-2B cells by protein extraction kit (Wanleibio). The concentration of protein was detected by BCA kit (Wanleibio). The protein was separated by SDS-PAGE and transferred to PVDF membrane (Millipore). The membrane was blocked by non-fat milk and then incubated with primary antibodies for SLC27A3 (Proteintech), STAU1 (Affinity), ACPL2 (Bioss), RABL4 (Biorbyt), and β-actin (Wanleibio), respectively. After 12-h of incubation at 4°C, the membrane was washed, incubated by the secondary antibody, and visualized by CEL (Wanleibio). β-actin serves as an internal control.

### Real-Time PCR Assay

Total RNA was extracted from lung tissues and BEAS-2B cells by TRIpure (BioTeke). The RNA was reverse transcribed into cDNA using BeyoRT™ II M-MLV RNase H- (Beyotime). Real-time PCR assay was conducted on Exicycler 96 system (BIONEER) using SYBR Green (Solarbio). The results was calculated using 2^-△△CT^ method. The primer sequences are as follows. Mus musculus ACPL2: F, AATCGCTTCTTGGTGCTG; R, CTACGCTTGGAATGTTGC. Mus musculus RABL4: F, GAAATGCTGGATAAGTTGTG; R, GAGGGAGATGCCTGAAGT. Mus musculus SLC27A3: F, GCATTGTGGGCTGCTTGG; R, GGGCTGGTTGACGAGGTAT. Mus musculus STAU1 F, GTAAAGAAACCAGGAGACG; R, CTGCTGATGGCTAAGATAA.

Mus musculus β-actin: F, CTGTGCCCATCTACGAGGGCTAT; R, TTTGATGTCACGCACGATTTCC. Homo sapiens ACPL2: F, ATGGAGCACTTCAAGGTAA; R, AGCAGAGTAGAGGGCAAA. Homo sapiens RABL4: F, GGAATGGATTTGGTGGTGAA; R, GAGATGCCTGGAGCCTGTGA. Homo sapiens SLC27A3: F, GTTCGGATGGCAAATGAGG; R, TGTACCGGGCAGTTGTGAG. Homo sapiens STAU1 F, ATCCGATTAGCCGACTGG; R, ACTTGAGTGCGGGTTTGG. Homo sapiens β-actin: F, GGCACCCAGCACAATGAA; R, TAGAAGCATTTGCGGTGG.

### ELISA Assay

The concentration of IL-6, IL-1β, and TNFα was measured by ELISA. ELISA kits (Wanleibio) for IL-6, IL-1β, and TNFα were used in this study and conducted according to the manufacturer’s instructions. The optical density was measured by a microplate reader (BIOTEK).

### Statistical Analysis

All statistical analyses were performed in R (version 3.6.1). The nonparametric Kruskal-Wallis test was used to perform intergroup comparisons of continuous variables. The degree of efficacy of each diagnostic biomarker was assessed using receiver operating characteristic (ROC) curves. Pearson correlation was used to analyze the relationship between the expression of diagnostic biomarkers and infiltrating immune cells. Comparison between two groups or among multiple groups were analyzed by student t-test or one-way ANOVA, respectively. Statistical significance was identified based on *P* < 0.05. Furthermore, bioinformatics analysis almost run for two months.

## Results

### Screening of DEGs in COPD

**Figure 1:**
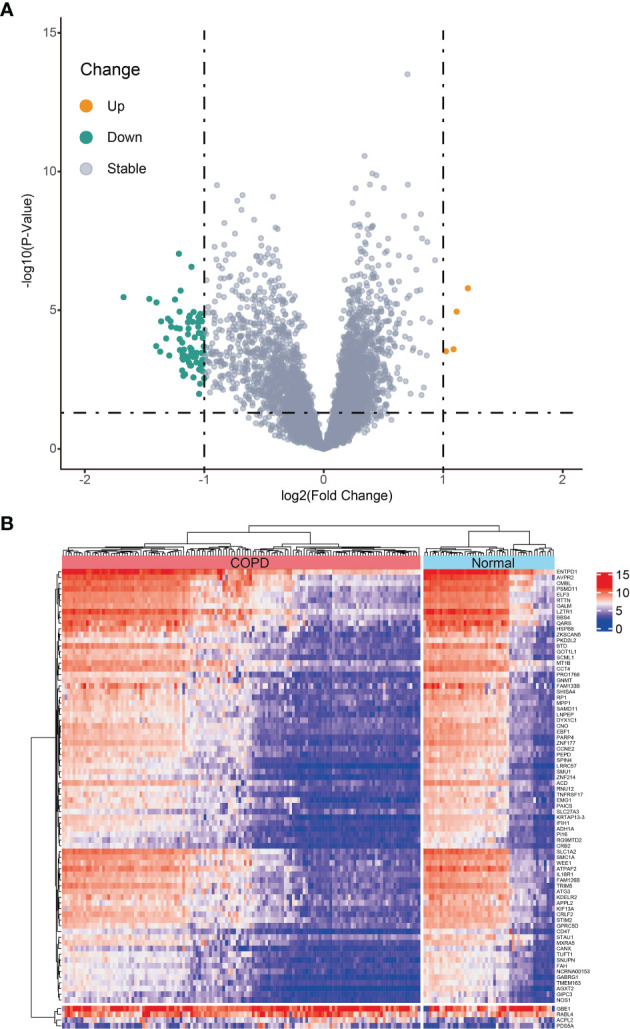
Differentially expressed genes (DEGs) between chronic obstructive pulmonary disease tissues and normal samples in metadata (GSE38974 and GSE76925 datasets) cohort. **(A)** Volcano plot of DEGs; orange dots represent up-regulated differential genes, gray dots represent nonsignificant genes, and green dots represent down-regulated differential genes. **(B)** Heatmap plot of DEGs.

Differential expression analysis between 134 COPD samples and 49 normal samples in the metadata (GSE38974 and GSE76925) cohort was carried out utilizing the “limma” R package. Of the 80 identified DEGs, 4 genes were significantly upregulated and 76 genes were significantly downregulated (**Figure 1A**). The heatmap of DEGs is depicted in **Figure 1B**.

### Functional Enrichment Analysis of DEGs

**Figure 2:**
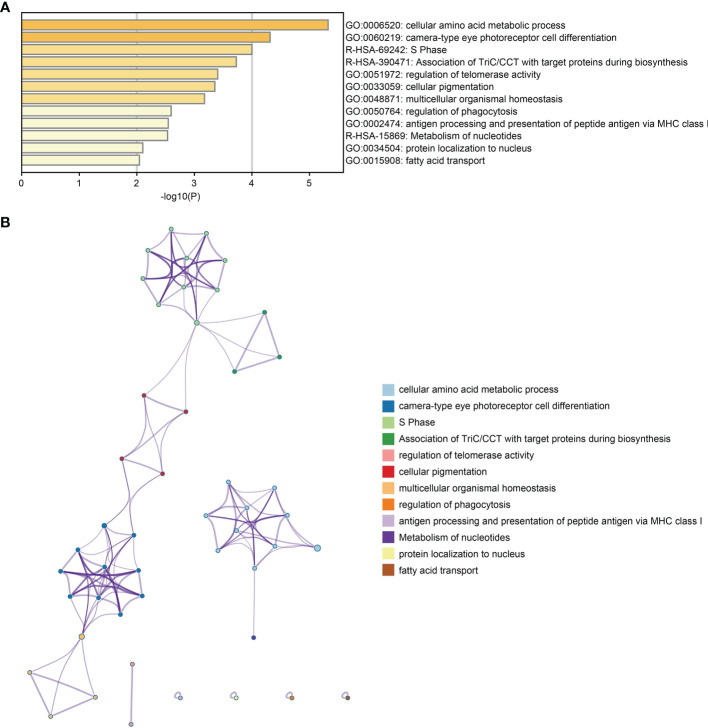
Functional enrichment analysis of DEGs. **(A)** Bar plot of DEGs functional enrichment terms. **(B)** Network relationship plots among all terms.

GO and pathway analyses were performed to identify the biological functions of DEG using the Metascape database. DEGs were primarily involved with telomerase regulation, cellular amino acid metabolic processes, multicellular organic homeostasis, MHC class I-mediated peptide antigen presentation and antigen processing in addition to various other biological processes (**Figure 2A**). Moreover, these DEGs were also mainly enriched in the S phase, TriC/CCT association with target proteins during biosynthesis and nucleotide metabolism pathways. **Figure 2B** shows the relationships between the enriched terms.

### Identification and Verification of Diagnostic Biomarkers

**Figure 3:**
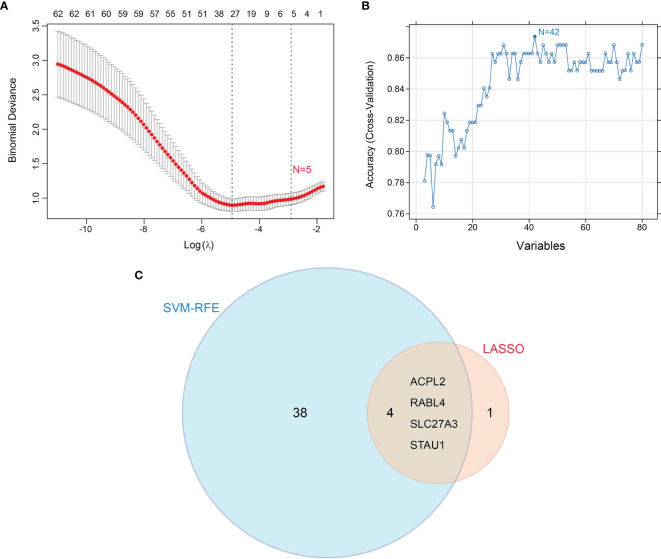
Screening candidate diagnostic markers for chronic obstructive pulmonary disease**. (A)** Diagnostic markers were screened by the least absolute shrinkage and selection operator (LASSO) logistic regression algorithm. **(B)** Diagnostic markers were screened by a support vector machine-recursive feature elimination (SVM-RFE) algorithm. **(C)** Venn diagram of variables screened by LASSO and SVM-RFE algorithms.

**Figure 4:**
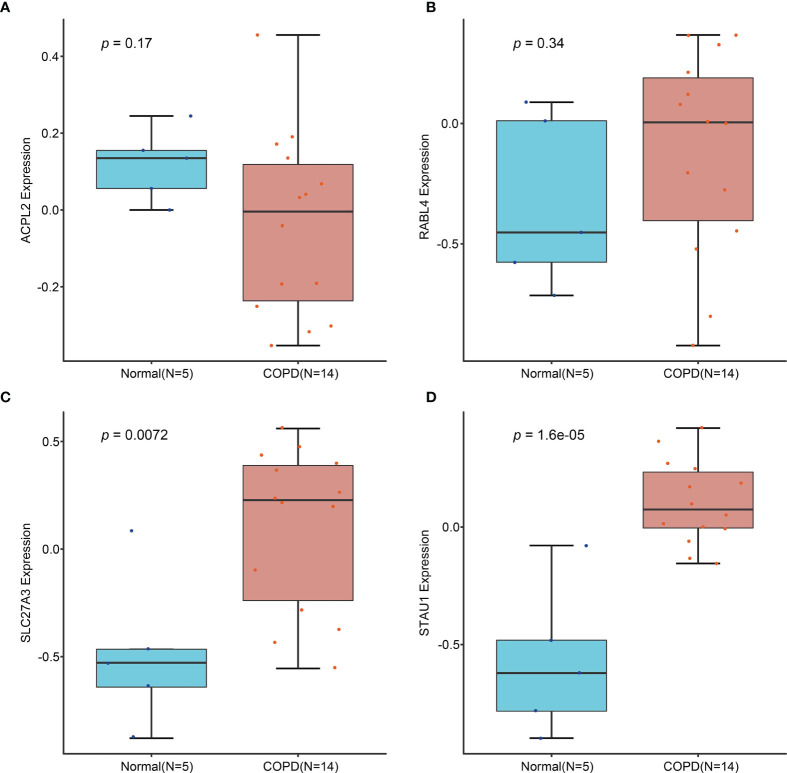
Validation of the expression of candidate diagnostic markers in the GSE106986 dataset. **(A)** ACPL2; **(B)** RABL4; **(C)** SLC27A3; **(D)** STAU1.

**Figure 5:**
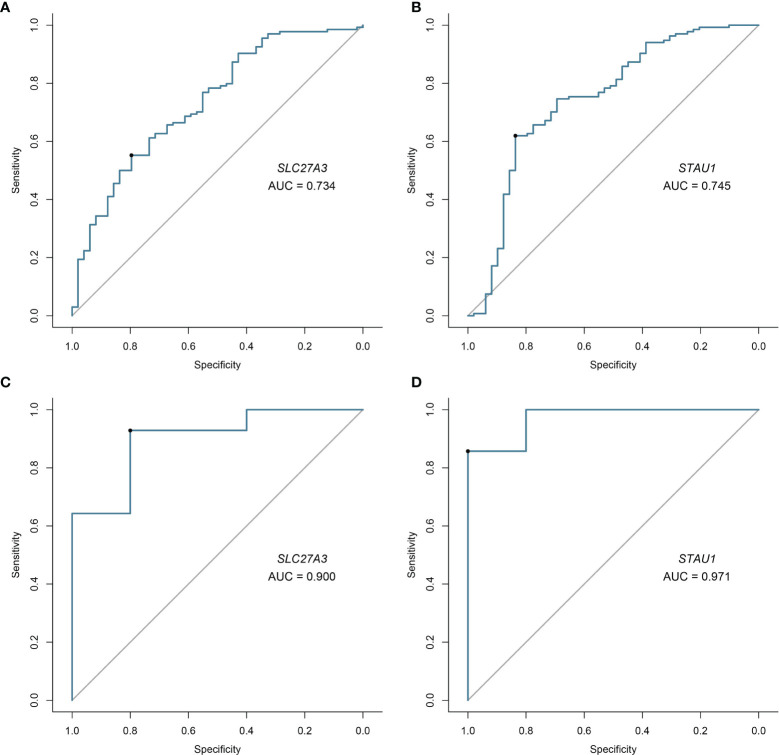
Validation of diagnostic validity of two diagnostic markers. **(A, B)** Receiver operating characteristic (ROC) curves of SLC27A3 and STAU1 in the metadata cohort. **(C, D)** ROC curves of SLC27A3 and STAU1 in the GSE106986 dataset.

Candidate diagnostic biomarkers were screened by two different algorithms. We utilized the LASSO logistic regression algorithm to identify and COPD related 5 feature variables from DEGs (**Figure 3A**). The SVM-RFE algorithm was used to identify a subset of 42 features in the determined DEGs (**Figure 3B**). 4 overlapping diagnostic related genes (ACPL2, RABL4, SLC27A3, and STAU1) of these two algorithms were finally selected (**Figure 3C**). The GSE106986 dataset was used to validate the accuracy of this method as well as the expression levels of the four candidate diagnostic biomarkers. ACPL2 and RABL4 expressions levels were not significantly different between COPD and normal samples (**Figures 4A, B**). However, the expression levels of SLC27A3 and STAU1 in COPD samples were notably raised in COPD samples in contrast to controls (**Figures 4C, D**; all *P* < 0.05). To further test the diagnostic efficacy of SLC27A3 and STAU1, we validated them using metadata and the GSE106986 dataset, respectively. The AUCs of SLC27A3 and STAU1 in the metadata cohort were 0.734 and 0.745, respectively (**Figures 5A, B**). Furthermore, the AUCs of SLC27A3 and STAU1 in the GSE106986 dataset were 0.900 and 0.971, respectively (**Figures 5C, D**), indicating that both SLC27A3 and STAU1 have high diagnostic values.

### Immune Cell Infiltration Landscape

**Figure 6:**
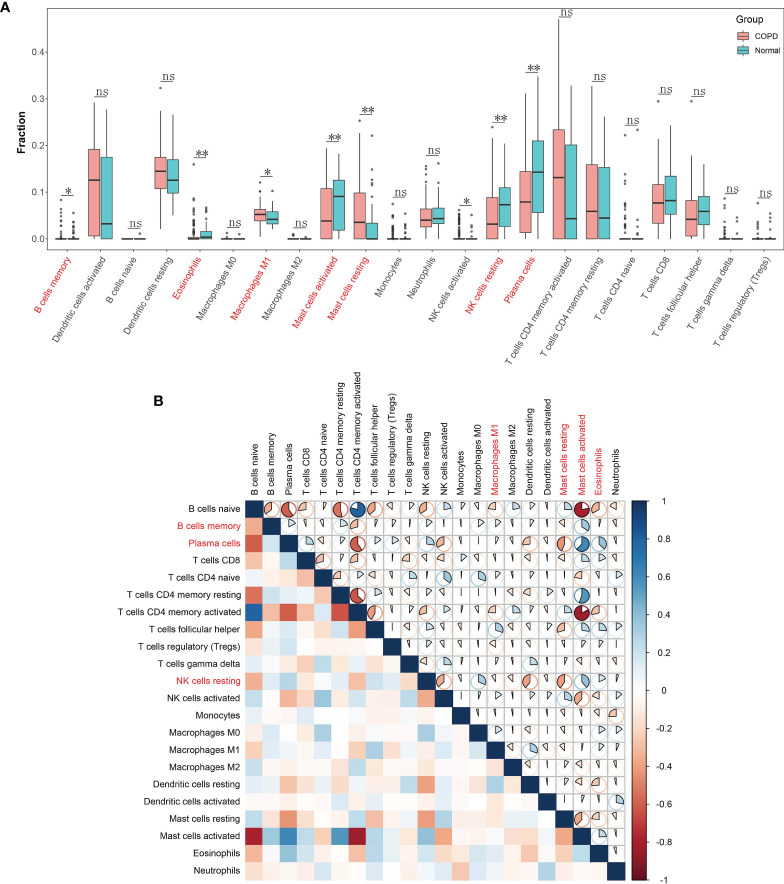
Landscape of immune cell infiltration in chronic obstructive pulmonary disease. **(A)** Boxplot of the proportion of 22 types of immune cell infiltrates. Red in the boxes represents the chronic obstructive pulmonary disease group and blue represents the normal group. *P < 0.05; **P < 0.01; ns, not significant. **(B)** Heatmap plot of correlations among 22 types of immune cells. Blue and red indicate positive and negative correlation, respectively. The darker the color the stronger the correlation.

With the CIBERSORT algorithm, we firstly calculated the proportion of immune cell infiltration in COPD and normal tissues. The results showed that the degree of infiltration of resting mast cells (*P* = 0.001), M1 macrophages (*P* = 0.049), and memory B cells (*P* = 0.020) in COPD tissues were notably raised in COPD samples in contrast to controls. Conversely, control samples demonstrated a higher proportion of infiltration of eosinophils (*P* = 0.002), activated mast cells (*P* = 0.006), resting NK cells (*P* = 0.004) and plasma cells (*P* = 0.003) compared to those found in COPD tissues (**Figure 6A**). Furthermore, we calculated the correlation between the 22 types of infiltrating immune cells (**Figure 6B**). Plasma cells and resting NK cells both individually correlated negatively with resting mast cells but positively with activated mast cells. Moreover, activated mast cells correlated positively with memory B cells.

### Correlation Analysis Between SLC27A3, STAU1 and Infiltrating Immune Cells

**Figure 7:**
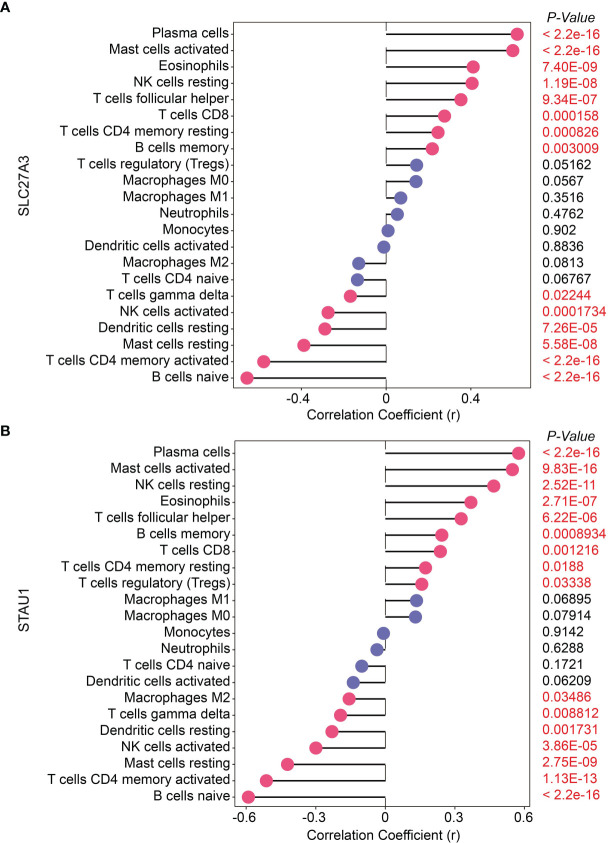
Correlation of two diagnostic markers with 22 types of immune cell infiltration in chronic obstructive pulmonary disease. **(A)** SLC27A3; **(B)** STAU1.

SLC27A3 was significantly positively correlated with memory B cells (*r*=0.218, *P*=0.003), CD4 memory resting T-cells (*r*=0.245, *P*<0.001), CD8 T-cells (*r*=0.276, *P*<0.001), follicular T-helper cells (*r*=0.353, *P*<0.001), resting NK cells (*r*=0.406, *P*<0.001), eosinophils (*r*=0.411, *P*<0.001), activated mast cells (*r*=0.598, *P*<0.001) and plasma cells (*r*=0.619, *P*<0.001), and significantly negatively correlated with naive B cells (*r*=-0.657, *P*<0.001), CD4 memory activated T-cells (*r*=-0.578, *P*<0.001), resting mast cells (*r*=-0.388, *P*<0.001), resting dendritic cells (*r*=-0.289, *P*<0.001), activated NK cells (*r*=-0.274, *P*<0.001) and gamma delta T-cells (*r*=-0.169, *P*=0.022; **Figure 7A**). STAU1 was significantly positively correlated with regulatory T-cells (*r*=0.157, *P*=0.033), CD4 memory resting T-cells (*r*=0.174, *P*=0.019), CD8 T-cells (*r*=0.237, *P*=0.001), memory B cells (*r*=0.244, *P*<0.001), follicular T-helper cells (*r*=0.327, *P*<0.001), eosinophils (*r*=0.369, *P*<0.001), resting NK cells (*r*=0.468, *P*<0.001), activated mast cells (*r*=0.548, *P*<0.001) and plasma cells (*r*=0.575, *P*<0.001), and significantly negatively correlated with naive B cells (*r*=-0.590, *P*<0.001), CD4 memory activated T-cells (*r*=-0.513, *P*<0.001), resting mast cells (*r*=-0.422, *P*<0.001), resting dendritic cells (*r*=-0.230, *P*=0.002), activated NK cells (*r*=-0.299, *P*<0.001), gamma delta T-cells (*r*=-0.193, *P*=0.009) and M2 macrophages (*r*=-0.156, *P*=0.035; **Figure 7B**).

### Immune Cell Infiltration in COPD Mouse Models

**Figure 8:**
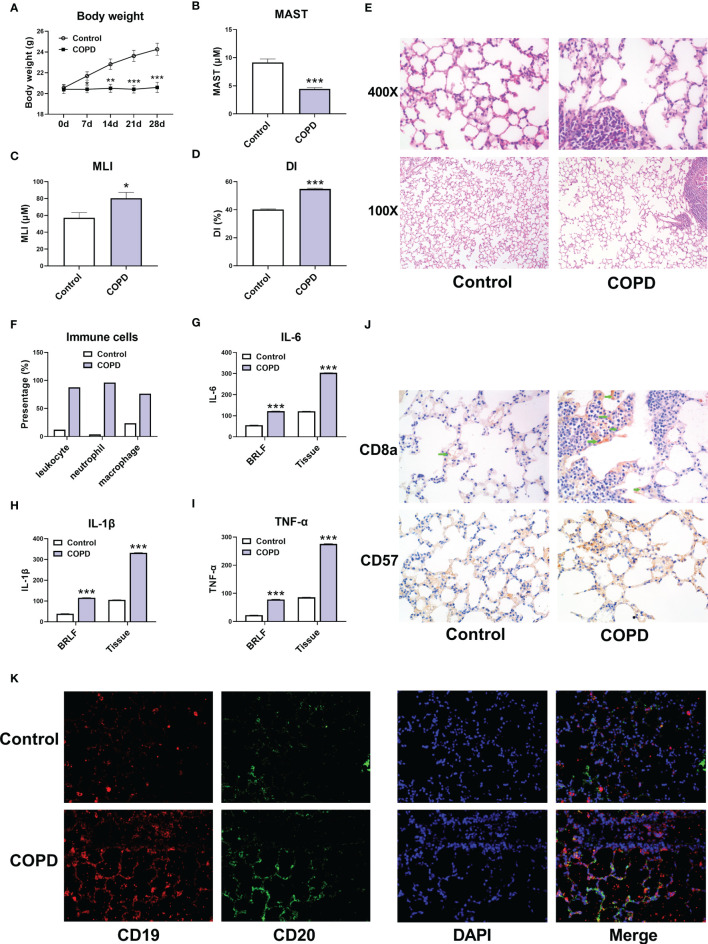
Immune cell infiltration in COPD mouse models. **(A)** Body weight of mice. **(B)** The mean alveolar septal thickness (MAST). **(C)** Mean linear intercept (MLI). **(D)** Destructive index (DI). **(E)** Representative HE staining images (100x and 400x). **(F)** The proportion of immune cells. **(G)** The expression of IL-6 in bronchoalveolar lavage fluid (BRLF) and lung tissues of mice. **(H)** The expression of IL-1β in BRLF and lung tissues of mice. **(I)** The expression of TNF-α in BRLF and lung tissues of mice. **(J)** Representative immunohistochemistry images of CD8a and CD57 (400x). **(K)** Representative immunofluorescence images of CD19 and CD20 (400x). For panel G, H, and I, the unit for BRLF is pg/mL, and the unit for lung tissue is pg/mg. *P < 0.05, **P < 0.01, ***P < 0.001 vs. Control.

Subsequently, we established a COPD mouse model using GSE. The body weight of mouse in COPD group was significantly lower than that in control group (**Figure 8A**). HE staining showed that the MAST was shorter, and the MLI was larger in COPD group compared with that in control group (**Figures 8** **B–E**). We then observed the immune cell infiltrating in COPD group. The proportion of leukocyte, and the percentage of neutrophil and macrophage were larger in COPD group (**Figure 8F**). Simultaneously, the expression levels of IL-6, IL-1β, and TNF-α were all increased in bronchoalveolar lavage fluid (BRLF) and lung tissues from COPD group (**Figures 8** **G–I**). In addition, the expression of immune cell markers including CD8a, CD57, CD19, and CD20 were upregulated in lung tissues from mouse in COPD group (**Figures 8J, K**).

### The Expression of SLC27A3 and STAU1 in COPD Models

**Figure 9:**
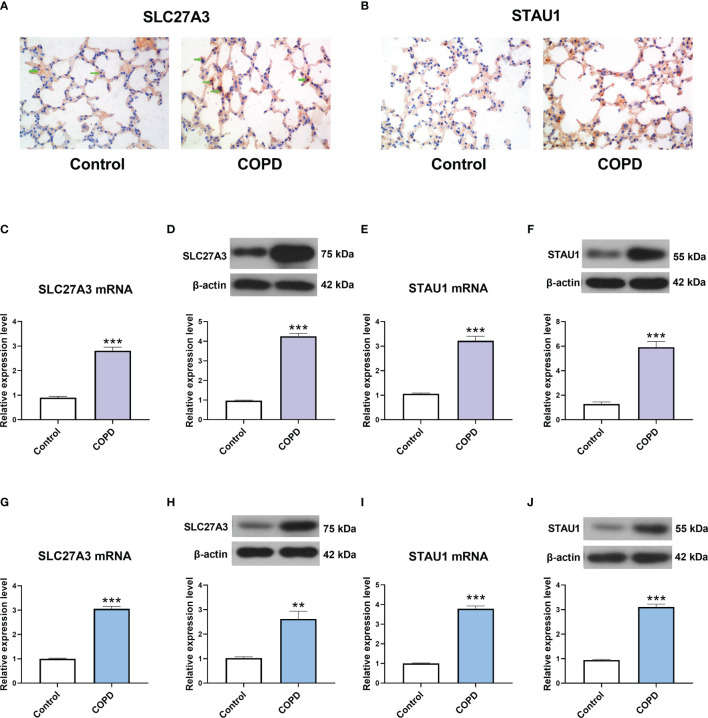
The expression of SLC27A3 and STAU1 in COPD models. **(A)** Representative immunohistochemistry images of SLC27A3 in lung tissues of mice. **(B)** Representative immunohistochemistry images of STAU1 in lung tissues of mice. **(C)** The expression of SLC27A3 mRNA in lung tissues of mice. **(D)** The expression of SLC27A3 protein in lung tissues of mice. **(E)** The expression of STAU1 mRNA in lung tissues of mice. **(F)** The expression of STAU1 protein in lung tissues of mice. **(G)** The expression of SLC27A3 mRNA in BEAS-2B cells. **(H)** The expression of SLC27A3 protein in BEAS-2B cells. **(I)** The expression of STAU1 mRNA in BEAS-2B cells. **(J)** The expression of STAU1 protein in BEAS-2B cells. **P < 0.01, ***P < 0.001 vs. Control.

GSE-induced COPD models were established both *in vivo* and *in vitro*. Immunohistochemistry staining, real-time PCR and western blot assay showed that the mRNA and protein levels of SLC27A3 and STAU1 were upregulated in lung tissues of mice from COPD group (**Figures 9** **A–F**). In accordance with these results, SLC27A3 and STAU1 were upregulated in GSE-treated BEAS-2B cells (**Figures 9** **G–J**). Besides, ALCPL2 was also upregulated in COPD models, while RABL4 was not significantly changed (**Figure S1**).

### Knockdown of SLC27A3 and STAU1 Reversed the Effect of CSE on BEAS-2B Cells

**Figure 10:**
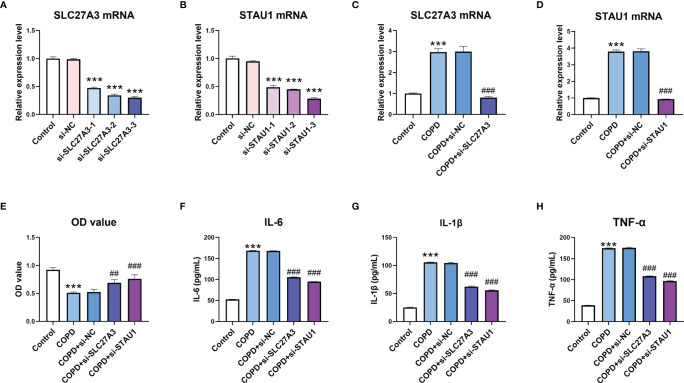
Knockdown of SLC27A3 and STAU1 reversed the effect of cigarette smoke extract (CSE) on BEAS-2B cells. **(A)** The effect of si-SLC27A3 on SLC27A3 mRNA expression in BEAS-2B cells. **(B)** The effect of si-STAU1 on SLC27A3 mRNA expression in BEAS-2B cells. **(C)** The expression of SLC27A3 mRNA in CSE-treated BEAS-2B cells after knockdown of SLC27A3. **(D)** The expression of STAU1 mRNA in CSE-treated BEAS-2B cells after knockdown of STAU1. **(E)** The viability of CSE-treated BEAS-2B cells after knockdown of SLC27A3 or STAU1. **(F)** The expression of IL-6 in CSE-treated BEAS-2B cells after knockdown of SLC27A3 or STAU1. **(G)** The expression of IL-1β in CSE-treated BEAS-2B cells after knockdown of SLC27A3 or STAU1. **(H)** The expression of TNF-α in CSE-treated BEAS-2B cells after knockdown of SLC27A3 or STAU1. For panel A and B, ***P < 0.001 vs. si-NC; for panel C-H, ***P < 0.001 vs. Control; ^##^P < 0.01, ^###^P < 0.001 vs. COPD+si-NC.

Finally, we observed the effect of SLC27A3 and STAU1 knockdown on CSE-treated BEAS-2B cells. There siRNAs sequences were designed to downregulate SLC27A3 and STAU1, respectively. The expression of SLC27A3 and STAU1 were significantly downregulated by transfection of corresponding siRNAs (**Figures 10** **A–D**). CSE treatment decreased the viability and increased the expression inflammatory cytokines (IL-6, IL-1β, and TNF-α) in BEAS-2B cells, which were attenuated by knockdown of SLC27A3 or STAU1 (**Figures 10** **E–H**).

## Discussion

COPD remains the third leading cause of death globally, with over three million dying annually from this debilitating disease despite significant advancements in diagnostic and therapeutic options over the last decade (24). Diagnosing COPD early is virtually impossible given the paucity of valuable biomarkers, a phenomenon that further results in poor clinical outcomes. Recent literature demonstrates that immune cell infiltration appears to be intricately involved in the occurrence and progress of COPD (25–27). Therefore, seeking for effective and novel diagnostic biomarkers for COPD amongst immune cell components is a promising avenue of research that may potentially result means of intervening early in COPD, thereby improving clinical prognosis. Recently, differentially expressed genes have been regarded as promising biomarkers in respiratory diseases, and especially in regards to COPD. For instance, HIF-1α could upregulate the expression of inflammatory factors, further aggravating the pathological process of COPD (28). In addition, FHL1 was found to be significantly modulated in CSE-treatment Beas-2B cells, providing important information on the role of inflammatory factors in COPD (14). However, there are few studies on biomarkers comprising of abnormally expressed genes associated with COPD and immune infiltration in normal tissues. Therefore, the purpose of this study is to search for candidate diagnostic biomarkers for COPD and explore how immune infiltration influences the progress of COPD.

This study is a novel retrospective report that comprehensively explores GEO datasets for likely diagnostic biomarkers amongst DEGs and immune cell infiltration in COPD patients. A metadata cohort was first formed by merging two GEO datasets which was then used to identify DEGs between COPD and control samples. Our study identified 4 significantly upregulated and 76 notably downregulated genes. Subsequent enrichment analysis found that these DEGs were primarily associated with cellular amino acid metabolic process, telomerase activity, phagocytosis regulation, MHC class I-related peptide antigen presentation and antigen processing, along with other biological processes. Moreover, DEGs were also enriched in the S phase and nucleotide metabolism. These findings are consistent with previously discovered factors related to COPD. Earlier experiments have detected altered amino acid levels in patients with serve COPD which may be helpful in the diagnosis and treatment of COPD (29, 30). Chronic inflammation is a prominent feature in COPD that results to airway remodeling and lung parenchyma destruction (31). The abnormalities in telomere function contributed significantly to sustained local inflammation in the lungs as well as systemic inflammation in COPD patients (32). COPD alveolar macrophages have defective phagocytic functions with regards to the uptake and processing of respiratory pathogens and cellular debris (33). Research shows that cigarette smoke can result in an increased percentage of human fetal lung fibroblasts cells in G1 and G2/m phases in addition to a reduced percentage of cells in S phase of cell cycle. Therefore, changes in the cell cycle potentially impact COPD pathogenesis (34). Immune cells have been proven to carry out an extremely vital role in COPD, and changes in the MHC I surface expression and MHC I-mediated presentation of a specific antigen caused by cigarette smoke may lead to a distorted adaptive immune response in viral and bacterial exacerbations of COPD patients (31, 35). These findings are consistent with our experimental results, suggesting that our discoveries are accurate as well as further solidifying the critical role of the immune response in COPD. Therefore, precise control of various types of immune cells remains a promising means of developing novel COPD treatment that may offer improved clinical prognosis. Bioinformatics analysis enables identification of significant novel COPD biomarkers associated with the degree of immune cell infiltration that may be beneficial for early diagnosis and treatment of COPD.

Our study identified two diagnostic markers *via* machine-learning algorithms. Staufen 1 (STAU1) is an RNA-binding protein that is related to post transcriptional mRNA regulation and is critical in cellular defense mechanisms against stressful stimuli such as oxidative and endoplasmic reticulum (36). Dysregulated STAU1-mediated post-transcriptional genetic modulation appears to regulate apoptosis while also being a pivotal occurrence in cancer progression (37, 38). Studies have revealed that STAU1 selectively regulates genes related to inflammation and immune responses and plays a substantial role in the human immune response against the influenza virus and human immunodeficiency virus type I (HIV-1) (39–42). These facts further underscore the vital function of STAU1 in inflammation and immunity. The solute carrier 27A (SLC27A) gene belongs to a family of genes that encodes fatty acids transport proteins (FATPs). FATPs are located in the cell membrane and intracellular space and are mainly related to fatty acid activation and absorption (43). The encoding product of SLC27A3 gene (FATP3) plays an important role in long-chain fatty acids transport and very-long-chain fatty acids activation, the upregulation of which could promote lipid metabolism (44). COPD pathogenesis has been found to be closely related to altered lipid metabolism (45). As a result, we infer that SLC27A may function to critically modulate the initiation and progression of COPD.

The CIBERSORT assessment was used in the study to determine the profiles of immune cell infiltration in COPD subjects and in healthy controls. A myriad of immune cell subtypes has been characterized in relation to COPD. Increased infiltration of memory B cells, M1 macrophages, and resting mast cells were found in COPD in contrast to normal tissues. However, proportions of eosinophils, activated mast cells, resting NK cells, and plasma cells were found to be decreased. Furthermore, the correlation analysis of STAU1, SLC27A3, and immune cells indicated that STAU1 and SLC27A3 correlated to plasma cells, activated mast cells, eosinophils, resting NK cells, CD8+, CD4+, and helper follicular T-cells, as well as in resting memory B cells. We then established GSE-induced COPD models both *in vivo* and *in vitro*. Immune infiltration and inflammatory cytokines production were increased in COPD models. Enhanced expression of STAU1 and SLC27A3 were observed in COPD models both *in vivo* and *in vitro*. Knockdown of SLC27A3 and STAU1 reversed the effect of CSE on BEAS-2B cells. Moreover, knockdown of SLC27A3 and STAU1 reversed the effect of CSE on the viability and the expression inflammatory cytokines in BEAS-2B cells. These findings were in accordance with the bioinformatics analysis results.

Primary pathological changes of COPD mainly comprise of emphysema and chronic bronchitis. Continual airflow restriction leads to pulmonary ventilation dysfunction, which in turn leads to progressively deteriorating lung function (46). Inflammatory mechanisms lie at the core of COPD development. Various factors can lead to inflammatory cell infiltration and release of inflammatory mediators. Inflammatory cells subsequently produce destructive enzymes that lead to the progressive destruction of lung tissue in COPD (47, 48). Studies have also found elevated expressions of CD8+T-cells in COPD lung tissues. CD8+ T-cells primarily secrete IL-4 and IL-5 cytokines, both of which are implicated in lung parenchymal tissue damage that exacerbates the development of emphysema (49). Some studies have reported that NK cells can secrete cytotoxic mediators, such as granzyme B and perforin, which may play an important role in inducing lung cell apoptosis and thereby promoting emphysema (50). There is evidence demonstrating that NK cells exert cytotoxic effects on COPD lung epithelial cells and are enhanced by the transport of IL-LS by dendritic cells of the IL-I5 receptor subUnita (LL-15RA) (51). In addition, it has been confirmed that the level of memory B cells in the peripheral blood of smokers increases, and smoking in patients is also one of the main causes of COPD (52). The wealth of evidence supporting the direct or indirect effects of inflammatory cells on COPD pathogenesis are consistent with our study findings. It is of great interest for future studies to identify potential immune targets for COPD immunotherapy.

Our study is firstly limited by its retrospective design and lack of analysis of important clinical data. Our identified upregulated genes seen to be associated with immune infiltration in COPD here require further validation in cohorts with clinical data. Secondly, the size of our GSE106986 validation cohort is small. We stress that our results should be validated in larger cohorts to determine the reproducibility of the findings. Although the functions of the two biomarkers and immune cell infiltration in COPD were assessed using bioinformatics analysis and biological experiments, larger prospective studies are needed to verify our conclusions.

## Conclusion

To sum up, the present study strongly suggests that STAU1 and SLC27A3 are significant diagnostic biomarkers in COPD. Plasma cells, resting NK cells, activated mast cells, eosinophils, CD8+, CD4+, and helper follicular T-cells, as well as memory B-cells are important factors in COPD development. Mechanisms of immune infiltration should be focused on in the search of novel therapeutics for COPD and these immune cells are expected to be promising targets for immunotherapy in patients with COPD.

## Data Availability Statement

The datasets presented in this study can be found in online repositories. The names of the repository/repositories and accession number(s) can be found in the article/**Supplementary Material**.

## Author Contributions

Conceptualization, YZ and SJ. Software, RX, ML, and ZL. Writing—original draft preparation, YZ, XC, YH, and CS. Writing—review and editing, YJ and SJ. All authors contributed to the article and approved the submitted version.

## Funding

This work was funded by the National Science Foundation of China (81670028), the Major Program of Natural Science Foundation of Heilongjiang Province (ZD2016014), Harbin City Applied Technology Research and Development Project (2016RQXYJ116).

## Conflict of Interest

The authors declare that the research was conducted in the absence of any commercial or financial relationships that could be construed as a potential conflict of interest.

## Publisher’s Note

All claims expressed in this article are solely those of the authors and do not necessarily represent those of their affiliated organizations, or those of the publisher, the editors and the reviewers. Any product that may be evaluated in this article, or claim that may be made by its manufacturer, is not guaranteed or endorsed by the publisher.
