# Supplementary material for: Machine-Learning Algorithm-Based Prediction of Diagnostic Gene Biomarkers Related to Immune Infiltration in Patients With Chronic Obstructive Pulmonary Disease
Source: Front Immunol. 2022 Mar 8;13:740513. doi: 10.3389/fimmu.2022.740513 (PMC8957805; doi:10.3389/fimmu.2022.740513)
Supplement: Supplementary file 1 [file DataSheet_1.docx]

Supplementary Material

**
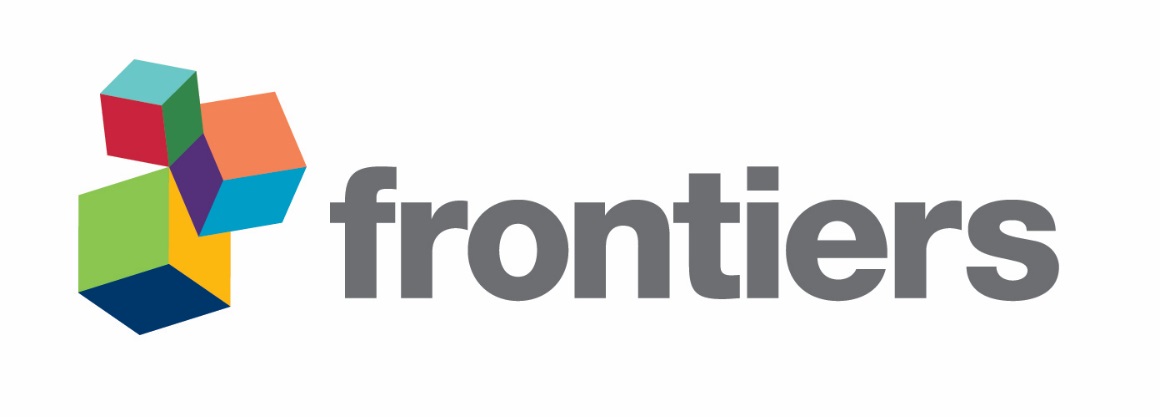
**


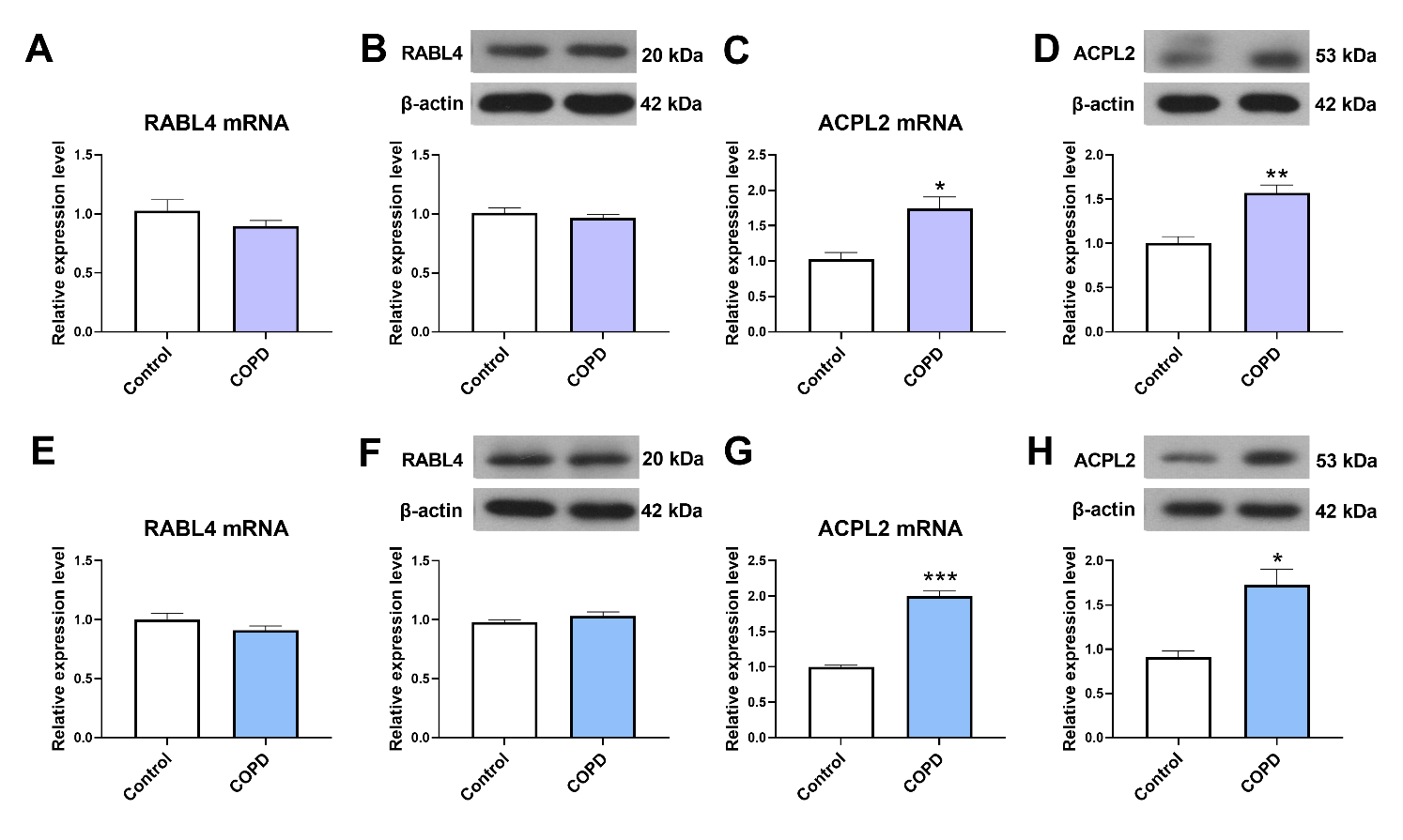


**Figure S1.** The expression of RABL4 and ACPL2 in COPD models. A. The expression of RABL4 mRNA in lung tissues of mice. B. The expression of RABL4 protein in lung tissues of mice. C. The expression of ACPL2 mRNA in lung tissues of mice. D. The expression of ACPL2 protein in lung tissues of mice. E. The expression of RABL4 mRNA in BEAS-2B cells. F. The expression of RABL4 protein in BEAS-2B cells. G. The expression of ACPL2 mRNA in BEAS-2B cells. H. The expression of ACPL2 protein in BEAS-2B cells. * P<0.05, ** P<0.01, *** P<0.001 vs. Control.
